# Nutrition and Supplementation Considerations to Limit Endotoxemia When Exercising in the Heat

**DOI:** 10.3390/sports6010012

**Published:** 2018-02-06

**Authors:** Joshua H. Guy, Grace E. Vincent

**Affiliations:** 1 School of Health, Medical and Applied Sciences, Central Queensland University, Cairns 4870, Australia; 2 School of Health, Medical and Applied Sciences, Central Queensland University, Adelaide 5034, Australia; g.vincent@cqu.edu.au

**Keywords:** heat stress, inflammation, cytokines, hydration, gastrointestinal permeability

## Abstract

Exercise-induced heat production is further elevated by exercise performed in hot conditions and this can subsequently impact inflammation, and gastrointestinal (GI) health. Implementing nutrition and supplementation strategies under these conditions may support the hyperthermic response, the systemic inflammatory response, GI permeability and integrity, and exercise performance. Therefore, the aim of this brief review is to explore athletes’ inflammatory response of two key biomarkers, lipopolysaccharide (LPS), and interleukin-6 (IL-6), and provide nutrition and supplementation recommendations when exercising in hot conditions. There is emerging evidence that probiotics, glutamine, and vitamin C can preserve GI integrity, which may improve performance during exercise in the heat. Glucose rich food when consumed with water, before and during exercise in the heat, also appear to limit endotoxemia, preserve GI integrity, and reduce the incidence of GI disturbances compared with water alone. The use of non-steroidal anti-inflammatory drugs (NSAIDs) may compromise GI integrity and this may result in greater leakage of endotoxins during long duration exercise in the heat. Further work is required to elucidate the impact of nutrition and supplementation strategies, in particular the use of NSAIDs, when exercising in the heat.

## 1. Introduction

The classical thermoregulatory model of heat stress has been well-documented [1] and previous research demonstrates that exercise-induced heat production is further elevated when exercise is performed in hot conditions [2]. However, the involvement of the thermoregulatory and inflammatory pathways in the regulation of heat strain and heat stroke is mixed. In sporting and occupational settings (e.g., firefighting), some individuals are able to physically perform adequately with elevated core temperatures, in some cases exceeding 40 °C [3]. In contrast, some athletes have demonstrated symptoms of heat stress at core temperatures below 40 °C [4]. Collectively, these findings suggest that hyperthermia is not the only driver of heat stress and heat illness. 

Field-based observations of individual susceptibility to heat stress/illness point to a more complex regulation involving both thermoregulatory and inflammatory processes [5]. There are two main inflammatory processes that occur in response to exercising in the heat, (1) an increase in the rise of circulating lipopolysaccharide (LPS), and (2) the release of cytokine interleukin-6 (IL-6). The rise of circulating LPS is associated with decreased splanchnic blood flow, exertional ischaemia, and increased gastrointestinal (GI) permeability as a result of exposure to hot conditions. IL-6, a pro- and anti-inflammatory cytokine and a myokine, is released during exercise in the heat manifesting mainly from the muscle [6]. Furthermore, the thermal stress experienced by athletes undertaking long duration exercise in the heat could trigger a systemic inflammatory response via interplay between these, and other mediators.

A commonly employed strategy to counteract the debilitating effects of heat on exercise performance is to undertake heat acclimation (e.g., in simulated environment, such as a heat chamber) or acclimatisation training (e.g., natural environment) [7]. Nevertheless, little research has considered the nutritional demands of exercising under hot environmental conditions. While a plethora of research has demonstrated benefits of nutrition and/or supplementation strategies for exercise performance in temperate conditions [8,9] there is sparse information on the utilisation of nutrition and/or supplementation strategies to alleviate the adverse thermoregulatory and inflammatory impacts when exercising in the heat. While there are numerous biological markers of heat stress and inflammation, this review will focus on the interaction between exercise in the heat, and nutrition and supplementation strategies, specifically in the context of GI disturbances and integrity, endotoxemia and inflammation. In particular the aforementioned biomarkers, (1) LPS and (2) the pro- and anti-inflammatory cytokine IL-6 will be discussed, as they directly influence the inflammatory cascade in response to heat stress and heat illness [10]. In turn, various nutrition and supplementation strategies may impact the concentration of these markers which may positively influence the hyperthermic response, the systemic inflammatory response, GI permeability and integrity, and ultimately exercise performance.

The contribution of the inflammatory pathway when exercising in hot conditions provides practitioners with valuable information on how to manage athletes under these circumstances. Further, this information also provides targets for possible nutrition and supplementation interventions to improve exercise performance and limit the degree of GI disturbances and permeability. It is therefore important to understand the degree to which circulating concentrations of biomarkers are affected by exercise in the heat, and the influence of various nutrition and supplementation strategies under these conditions. Therefore, the aim of this brief review is to examine athletes’ inflammatory response to exercising in the heat and explore potential nutrition and supplementation strategies that may alleviate heat stress and heat illness, and improve physical performance.

## 2. Lipopolysaccharide and Endotoxemia during Exercise

**Figure 1:**
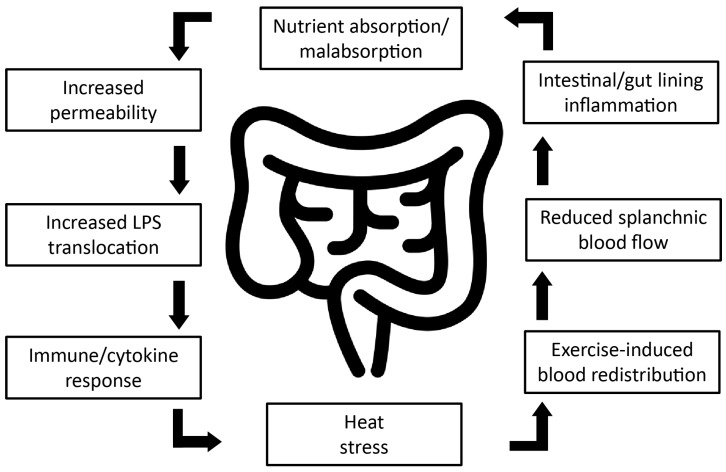
Diagrammatic representation of factors related to exercise induced gastrointestinal permeability. Strenuous physical exercise leads to redistribution of blood, shunting blood away from the splanchnic area, thereby significantly reducing splanchnic blood flow and resulting in mucosal damage and loss of integrity to the gut wall. Further research is required to determine whether exercise-induced mucosal injury can be attenuated by nutritional strategies, potentially increasing athletic performance, and improving post-exercise recovery. Adapted from Van Wijck et al., (2012) [13].

Increased thermoregulatory and cardiovascular strain during exercise in the heat is associated with redistribution of blood flow from internal organs to active skeletal muscle and peripheral tissues. The major impact of blood flow redistribution is disturbances to the GI epithelium [11] leading to increased GI permeability. Subsequently, LPS is released from Gram-negative bacteria residing in the gut, which is translocated to the portal system [10]. Elevated levels of LPS in the portal circulation can cause a dose-dependent systemic inflammatory response [11,12]. Further, high levels of LPS indicate that the GI mucosa is unable to fully preserve an effective barrier function, resulting in bacterial translocation from the gut lumen to the circulation [13]. When the rate of LPS clearance by the liver is overwhelmed by increased translocation after transient damage to the gut, endotoxemia can occur if there are insufficient levels of anti-LPS antibodies to aid in clearance [14]. A summary of the factors related to exercise induced gastrointestinal permeability are shown in Figure 1.

The role of LPS during strenuous exercise has been investigated since the 1980’s [15,16] with circulating LPS concentrations of >5 pg·mL^−1^ being indicative of mild endotoxemia [15,16,17]. Following an 8-h ultra-triathlon, mean circulating LPS concentrations increased to 294 pg·mL^−1^ [15]. Increases in LPS were also positively correlated with the incidence of cramping of the lower limbs [15]. In another early study, the majority (81%) of ultra-marathon runners had increased levels of LPS at the cessation of the race [16]. More recently, athletes exposed to consecutive days of exertional-heat stress during a multi-stage ultra-endurance marathon experienced a modest and sustained rise (21%, *p* < 0.001) in both resting and post-stage circulatory LPS [18]. Further, moderately-trained athletes have shown even greater rises (~50%) in circulating LPS during consecutive days of strenuous exercise in hot conditions [11]. However, athletes that have had more exposure to training at higher intensities suffer less from GI complaints, dehydration, and heat shock [15].

The relationship between GI complaints and gut ischaemia-associated leakage of LPS is not always clear. LPS leakage may be a relatively common feature of hard and sustained exercise, especially when cardiovascular and thermal strain is compounded by factors such as exogenous heat stress, upright posture, and dehydration [19]. Therefore, increased GI permeability, and the subsequent rise in circulating LPS, can exacerbate thermal strain by initiating an inflammatory cascade, perfusion abnormalities, and organ dysfunction [4]. These alterations in GI permeability are often expressed as increased concentrations of intestinal fatty acid binding protein (I-FABP) [13,19,20,21], lactulose to rhamnose ratio (L:R) [13,20,21,22,23], and lipopolysaccharide-binding protein (LPS-LPB) [24]. Following an Ironman distance triathlon, 68% of athletes exhibited an at least 150% increase in LPS [17]. However, while the majority (93%) of those athletes also reported GI symptoms, the severity of symptoms were not directly associated with endotoxemia (LPS > 5 pg·mL^−1^) [17]. This degree of endotoxemia is modest when compared to previous studies, for example, LPS levels above 100 pg·mL^−1^ were observed in 81% of athletes following an ultra-marathon [16]. Frequent exposure to tolerable concentrations of LPS during endurance races and training may lower plasma LPS response through enhanced LPS clearance mechanisms, such as anti-LPS antibodies and reticuloendothelial system activities [10]. Furthermore, individuals with lower aerobic fitness typically have a higher post-exercise plasma LPS concentration than more highly trained individuals when undertaking the same work [4]. When intolerable heat stress occurs [16], severe endotoxemia can ensue. Since endotoxemia is a balance of LPS influx and LPS clearance, this rate of clearance is likely to be a post-LPS translocation event. Although strenuous and/or extended competition in adverse environmental conditions can precipitate LPS translocation and endotoxemia, training to prepare for these types of events may also cause transient damage to the GI tract.

## 3. Cytokines

Cytokines can exert both pro-inflammatory and anti-inflammatory effects, and as such, act as both a mediator and protector in the resolution of inflammation [25]. However, the interaction between pro-inflammatory, anti-inflammatory, and immunoregulatory cytokines is complex and situation-specific. Furthermore, inflammatory responses are likely dependent on environmental conditions, exercise demands, and individual fitness levels [26]. A marked inflammatory response after heat stress is involved in both damage-generating processes, and recovery and repair mechanisms, following strenuous exercise. In normal circumstances, the inflammatory response after exercise is transient and diminishes quickly as homeostasis is re-established. However, under severe heat load, uncoupling of the regulatory balance between pro-inflammatory and anti-inflammatory cytokine responses is thought to exacerbate tissue damage [1].

Interleukin-6 plays an important role in the acute-phase response that is rapidly induced by inflammation associated with infection, injury and other factors. This reaction neutralizes pathogens and prevents their further invasion, and also minimises tissue damage [27]. The systemic effects of IL-6 appear to have a dose-response relationship with exercise duration and intensity. However, even though it is not unusual to see changes in IL-6 ranging from 1–100 fold, it is uncommon for exercise-induced peak plasma IL-6 concentration to exceed 100 pg·mL^−1^ [26]. In extreme athletic events, for example the 246 km “Spartathlon”, IL-6 increased 8000 fold [28]. Running at a 10% gradient at 65% of VO_2_ max for 40 min elevates circulating IL-6 by ~4 fold, as well as a moderate association (*r* = 0.67) with increases in core temperature during exercise in the heat [29]. Therefore, prior training that results in significant muscle damage, can influence the amount of IL-6 that will be released into circulation in subsequent exercise sessions. 

During multi-day events, such as an ultra-marathon, the preparation and ongoing health of the athlete is an obvious consideration. Throughout a five day ultra-marathon increases in IL-6 due to exertional-heat stress were observed, with levels continuing to be elevated following overnight recovery stages [18]. Increases in IL-6 were probably also related to the muscle damaging nature of the exercise performed on previous days. As a result, IL-6 responses were counteracted by compensatory anti-inflammatory cytokines that predominated throughout the ultra-marathon. Interestingly, the increases in IL-6 were not associated with GI symptoms that have been reported previously [17]. When exercise is performed with an additional heat stress or environmental load, the IL-6 response may be exacerbated due to increased GI permeability and the associated inflammatory assault. However, this outcome may not always be the case, and could be dependent on the athlete’s fitness level, individual ability to combat LPS, and the underlying health of their immune system. 

Some athletes appear to be susceptible to illness and infection during periods of increased training load, and exercise-induced IL-6 responses are higher in illness-prone athletes compared with healthy athletes (10 fold vs. 5 fold) [30]. Higher IL-6 concentration in illness-prone athletes following long distance running (60 min at 60% of VO_2_ max) could make athletes more susceptible to heat strain and illness due to impaired anti-inflammatory responses or poorly regulated cytokine balance. Therefore, athletes with susceptibility to illness may need to demonstrate additional caution when undertaking strenuous training blocks, particularly when being exposed to high heat loads. Further, the combination of mode, intensity and duration of the exercise likely determines the magnitude of the exercise-induced increase of plasma IL-6. Therefore, athletes may benefit from the implementation of nutrition and supplementation aimed at limiting the inflammatory response to exercise. 

## 4. Nutrition and Supplementation Strategies for Exercise in the Heat

Exercising in the heat can prompt heat stress and heat illness if preventative measures are not undertaken. A potential way to ameliorate the symptoms of heat stress and heat illness is by implementing nutrition and supplementation strategies. A summary of the gastrointestinal permeability and inflammatory cytokine response to exercise following nutrition and supplementation interventions are shown in Table 1.

*Vitamin C:* Given the role of LPS in the aetiology of endotoxemia, it is important to consider nutrition and supplementation interventions that may limit the influx of LPS by preserving GI integrity. Athletes may alter their response to heat stress and translocation of LPS via diet, supplementation, and the use of certain medications. Ascorbic acid is a naturally occurring compound with antioxidant properties, and supplementation could preserve luminal membrane integrity via antioxidant mechanisms [31]. Supplementation with ascorbic acid (a form of vitamin C) can reduce post-exercise LPS concentration by ~12 fold [31]. Conversely, the use of anti-inflammatory medications such as Ibuprofen may aggravate exercise-induced intestinal injury [13], thereby increasing the potential for GI leakage, leading to a greater influx of LPS into circulation. Therefore, the use of supplements to attenuate the rise in LPS following strenuous exercise may be beneficial for some athletes, but the use of anti-inflammatory agents such as Ibuprofen may further increase GI permeability.

*Probiotics:* Probiotic supplementation has been shown to reduce post-exercise LPS concentrations after running in hot conditions (35–40 °C) [22]. Although probiotic supplementation does not directly impact increased core temperature while exercising in the heat, decreases in GI permeability, as demonstrated by large reductions (15%) in post-exercise LPS translocation have been observed. These changes also translated to improvements to performance, shown by an increase in time to exhaustion during probiotic supplementation [22]. However, further studies are needed to determine the underlying mechanism behind improvements in performance following probiotic supplementation.

*Bovine Colostrum:* Other supplements such as bovine-colostrum have been suggested to curtail GI permeability via reduced apoptosis and paracellular permeability [32]. However, 8-weeks of bovine-colostrum supplementation has also been reported to increase GI permeability in recreational runners [23]. The increase in GI permeability with bovine-colostrum may have been related to greater leakiness of tight junctions between cells of the GI tract, or by increasing macromolecular transport, as occurs in the neonatal gut [23]. Alternatively, shorter periods (e.g., 1-week) of bovine-colostrum supplementation may have no influence on GI permeability and circulating concentrations of pro- and anti-inflammatory cytokines [19]. Therefore, any small benefits that may be achieved with bovine-colostrum supplementation may not benefit longer duration events such as a triathlon or ultra-endurance running, where there is likely greater internal and external heat load and storage, and a greater likelihood of GI permeability.

*Glutamine:* Glutamine is a natural non-essential amino acid which performs multiple roles, including acting as fuel for cells of the gut mucosa and immune system [33]. Previous research has demonstrated that acute oral glutamine consumption can attenuate GI permeability relative to placebo during a 60 min treadmill run at 70% VO_2_ max in hot environmental conditions (30 °C and ~40% relative humidity (RH)) [21]. However, it remains unknown whether the observed reductions in GI permeability will lead to reduced GI symptoms or heat induced endotoxemia [21].

*Carbohydrates and Protein:* When exercising in the heat, a commonly used strategy is the ingestion of carbohydrates (e.g., glucose), water, and less often protein [34]. Frequent ad libitum ingestion of glucose (15 g) before and during a 120 min treadmill run at 60% VO_2_ max in hot conditions (~35 °C and 27% RH) ameliorates intestinal epithelial injury, small intestine permeability, and enhances anti-endotoxin antibody responses, when compared to water alone [20]. Therefore, it may be beneficial to ingest glucose before and during exercise in the heat. Interestingly, energy-matched whey protein hydrolysate (15 g) also ameliorates intestinal epithelial injury and small intestine permeability, compared to water. However, whey protein ingestion increases the incidence and severity of GI symptoms compared to glucose and water [20]. This is in contrast to the aforementioned study that showed that glutamine reduced GI permeability but did not increase GI disturbance symptoms [21]. We speculate that the consistency of the ingested fluid, (i) glutamine dissolved in water with lemon cordial [21], and (ii) whey hydrolysed protein mixture [20] (which is characteristically denser), may contribute to the discrepancy in GI disturbance symptoms observed between these studies. Furthermore, following a short-course duathlon, a recent study reported higher dehydration levels and a mild increase in markers of hepatic damage in those with a low-carbohydrate diet compared to a high-carbohydrate diet. However, acute exercise-induced mild endotoxemia (increased LPS) was present in both dietary conditions and did not alter physical performance or IL-6 response [24].

Despite many studies detailing the acute responses of IL-6 to exercise in the heat, few studies have investigated the nutrition and supplementation strategies that may attenuate the IL-6 response. Increased heat load is known to elevate carbohydrate utilisation via accelerated glycogen breakdown as a result of increased core body temperature [35], and, greater circulation of catecholamines [26]. This process can result in the subsequent synthesis, signalling and release of IL-6. To counter the increased glycogen utilisation and the subsequent rises in IL-6 following exercise in the heat, one logical strategy would be to consume a greater amount of carbohydrate, thus increasing the availability of muscle glycogen and potentially attenuating the rise of IL-6. For example, high carbohydrate diets have been shown to have an attenuating effect [36,37] on the rise of IL-6 following exercise in temperate conditions. Therefore, further work is required to determine the effect of a high carbohydrate diet on the IL-6 response to exercise in the heat where muscle glycogen utilisation is substantially increased. 

*Precautionary strategies:* While various supplementation strategies have been investigated to maintain GI integrity under exercise induced heat stress, GI integrity can be compromised by over-the-counter anti-inflammatory medications [38]. Non-steroidal anti-inflammatory drugs (NSAIDs) are commonly used by athletes to reduce pain or prevent anticipated musculoskeletal pain during exercise [39,40]. The prevalence of NSAID usage has been reported in up to 90% of triathletes [41] and professional soccer players [38]. NSAIDs can aggravate GI injury during strenuous exercise, leading to the loss of gut barrier function in otherwise healthy athletes [13]. In addition, NSAIDs may promote splanchnic hypoperfusion, leading to the GI tract becoming further compromised. As endurance athletes experience significant GI injury after exercise without using NSAIDs [17,42], the combination of exercise and NSAIDs in scenarios where athletes undertake long-duration endurance exercise in the heat may increase the incidence of thermal injury or GI disturbances. While athletes undertaking endurance events often manage their nutrition and hydration to reduce the risk of heat stroke/stress, there is limited information surrounding the use of common NSAIDs in these circumstances.

### Other Nutrition and Supplementation Considerations

There are other nutrition and supplementation considerations when exercising in hot conditions that warrant attention. In particular, the influence of commonly used ergogenic aids can impact exercise heat tolerance and hydration status, and have been comprehensively reviewed elsewhere [43]. A summary of the recent literature pertaining to some of the more commonly utilised nutrition and supplementation strategies are summarised below. Although these studies may not have direct outcomes on exercise induced endotoxemia or inflammatory responses, the impact of these strategies for athlete management during exercise in the heat should be considered.

*Creatine:* a systematic review concluded that there was no evidence to support claims that creatine impedes heat tolerance and hydration status [44]. Further, there is some evidence to suggest that when exercising in the heat, creatine supplementation results in a lower body temperature compared to placebo [45,46,47], that may result in protective benefits against heat stress by maintaining haematocrit, aiding thermoregulation and reducing exercising heart rate and sweat rate [48]. 

*Caffeine:* Caffeine is one of the most commonly used drugs by athletes, due to its strong ergogenic effects. Several studies have noted little adverse effects of caffeine on thermoregulatory response, when compared to non-caffeinated sports drinks or placebo [49,50]. Further there is little evidence to support that chronic caffeine use leads to dehydration or heat intolerance [51]. Therefore, even though caffeine has mild diuretic properties, these are likely negated when fluid status is compromised during exercise in the heat, and thus there is little evidence to suggest avoiding caffeine. 

*Glycerol:* Hyperhydration prior to exercising in hot conditions can improve hydration status and increase heat tolerance. Hyperhydration with water alone directly increase urine output which can be ineffective. However, adding glycerol to water has been shown to promote hyperhydration (by as much as 50%) [52], decrease thermoregulatory strain, and improve exercise performance. For example, athletes exercised for 19% and 72% longer when hydration included glycerol ingestion, compared with water only and non-fluid trials, respectively [53]. In contrast, another study did not observe significant improvements in exercise performance or thermoregulation with glycerol ingestion during a 60 min self-paced cycling bout in 34.5 °C [54]. The discrepancy in the literature makes it difficult to determine whether hyperhydration with glycerol improves thermoregulation and performance when exercising in the heat. However, in certain situations, for example high-intensity exercise in the heat, or limited fluid access, pre-exercise glycerol ingestion may aid in maintaining hydration status [43]. 

*Hydration:* During intense exercise, fluid intake recommendations are at least 0.5 L·h^−1^, and likely greater in hot conditions [55]. Fluids that also include carbohydrate (<10%) or sodium are also encouraged to assist with both intestinal absorption of water and muscle glycogen replenishment, respectively [55]. However, it is important for athletes not to ingest beverages that have high carbohydrate concentrations as these may decrease fluid delivery to the gut, and increase GI underperfusion [56,57]. Therefore, it is also recommended to advise athletes about the dangers of drinking too much water [58], and to refrain from carbohydrate rich fluid replacements.

*Precautionary strategies:* While various supplementation strategies have been investigated to maintain GI integrity under exercise induced heat stress, GI integrity can be compromised by over-the-counter anti-inflammatory medications [38]. Non-steroidal anti-inflammatory drugs (NSAIDs) are commonly used by athletes to reduce pain or prevent anticipated musculoskeletal pain during exercise [39,40]. The prevalence of NSAID usage has been reported in up to 90% of triathletes [41] and professional soccer players [38]. NSAIDs can aggravate GI injury during strenuous exercise, leading to the loss of gut barrier function in otherwise healthy athletes [13]. In addition, NSAIDs may promote splanchnic hypoperfusion, leading to the GI tract becoming further compromised. As endurance athletes experience significant GI injury after exercise without using NSAIDs [17,42], the combination of exercise and NSAIDs in scenarios where athletes undertake long-duration endurance exercise in the heat may increase the incidence of thermal injury or GI disturbances. While athletes undertaking endurance events often manage their nutrition and hydration to reduce the risk of heat stroke/stress, there is limited information surrounding the use of common NSAIDs in these circumstances.

## 5. Recommendations and Future Research Directions

Practitioners and athletes should be aware of the impact of various nutrition and supplementation strategies that could affect GI integrity, inflammation, and performance when exercising in the heat. There is preliminary evidence supporting the use of pre-competition probiotic supplementation over several weeks to improve exercise performance. Further, acute vitamin C, and high-carbohydrate food (before and during exercise) appear to limit endotoxin leakage, and may attenuate rises in IL-6. Protein ingestion before and during exercise may increase the symptoms of GI disturbance; therefore, its use prior to exercise in the heat requires further investigation. It is crucial that nutrition and supplementation strategies be implemented into training programs prior to competition to reduce the likelihood of GI disturbance. Athletes should also limit their intake of NSAIDs during exercise in the heat, as this may exacerbate GI permeability and result in increased levels of LPS and inflammation. 

This brief review has identified scenarios in which common nutrition and supplementation strategies may aid in maintaining GI integrity and limit the inflammatory response as a result of exercise induced heat stress. The impact of nutrition and supplementation on athletic performance, with particular reference to the ability to attenuate GI permeability during intense exercise, remain poorly understood. Further, research is needed to clarify the mechanisms of action and effectiveness of nutrition and supplementation strategies under different environmental conditions. For example, a series of randomised controlled trials investigating the influence of various nutrition and supplementation strategies would provide important information on hydration, thermoregulation, and performance when exercising in the heat. In addition, work is required to determine safe practice of NSAIDs during long duration events (e.g., Ironman triathlon). Furthermore, little research has explored the effects of permissive dehydration on endotoxemia or the inflammatory response to exercise in the heat, and whether or not this improves exercise performance or places the athletes at a higher risk of a heat-related illness.

## 6. Conclusions

The past decade has seen the emergence of new models and insights into thermoregulation during exercise and causes of heat illness. It is now recognised that inflammatory pathways can also contribute to heat illness in a variety of settings, and there appears to be direct interplay between GI leakage of LPS and inflammatory cytokines such as IL-6. Therefore, athletes training and competing in hot environments should consider how nutrition and supplementation strategies influence the inflammatory response. Given the majority of research is focused on the acute effects of either races or one-off bouts of exercise, it is important to consider the cumulative effect of long-term nutrition or supplementation interventions and how that may affect GI integrity and limit exercise induced inflammatory response.

## Figures and Tables

**Table 1:** Gastrointestinal permeability and inflammatory cytokine response to exercise following nutrition and supplementation interventions.

Author	Oxygen Uptake (mL·kg^−1^·min^−1^) and Sample Size (n)	Experimental Conditions	Exercise and Nutrition/Supplementation Intervention	Biomarker Response
Ashton et al., (2003) [31]	49 ± 3, *n* = 10	Laboratory (temperate)	1000 mg of l-ascorbic acid (vitamin C) 2 h before exercise. Incremental cycle test to exhaustion.	L-ascorbic acid: ↓ LPS
Bishop et al., (2001) [36]	49 ± 3, *n* = 7	Laboratory (22 °C, 56% RH)	3 day Low-CHO or High-CHO diet. 60 min cycle at 60% Wmax and TT	High-CHO: ↓ IL-6 Low-CHO: ↑ IL-6
Buckley et al., (2009) [23]	53 ± 2, *n* = 30	Laboratory (temperate)	8 week daily supplementation 60 g Bovine Colostrum. Running 3 times per week for 45 min at lactate threshold.	Bovine Colostrum: ↑ L:R
Cox et al., (2010) [37]	65 ± 5, *n* = 16	Laboratory (temperate)	28 day Moderate-CHO or High-CHO diet. 100 min steady state cycling at 70% VO_2_ max and ~30 min TT.	Moderate-CHO: ↑ IL-6 High-CHO: ↑ IL-6,
Moncada-Jiménez et al., (2010) [24]	57 ± 7, *n* = 11	Laboratory (temperate)	48 h Low-CHO or High-CHO. Duathlon, 5 km run, 30 min stationary cycle, 10 km run.	Low-CHO: ↑ IL-6 and LPS-LPB High-CHO: ↑ IL-6 and LPS-LPB
Morrison et al., (2014) [19]	64 ± 4, *n* = 7 46 ± 4, *n* = 8	30 °C, 50% RH	1 week daily supplementation 1.7 g·kg^−1^ Bovine Colostrum. 30 min cycling at 50% HRR, 30 min running at 80% HRR	Bovine Colostrum: ↑ IL-6 and I-AFBP
Shing et al., (2014) [22]	63 ± 6, *n* = 10	35 °C, 40% RH	4 weeks daily supplementation probiotics capsule. Running at to exhaustion at 80% of ventilatory threshold	Probiotic: ↓ L:R and LPS Probiotic and Placebo: ↑ IL-6
Pugh et al., (2017) [21]	52 ± 5, *n* = 10	30 °C, 40–45% RH	0.25, 0.5 or 0.9 g·kg^−1^ glutamine 2 h before exercise. 60 min treadmill run at 70% of VO_2_ max	0.25, 0.5 and 0.9 g.kg^−1^ ↓ L:R 0.5 and 0.9 g.kg^−1^ ↓ I-AFBP
Snipe et al., (2017) [20]	54 ± 6, *n* = 11	35 °C, 27% RH	Water or CHO (15 g) or energy-matched PRO before and every 20 min during 2 h running at 60% VO_2_ max	CHO and PRO: ↓ I-AFBP and L:R CHO: ↓ IL-6 and LPS
Van Wijck et al., (2012) [13]	Well trained, *n* = 9	Laboratory (temperate)	400 mg ibuprofen 1 h before exercise. Cycling at 70% Wmax, ↓ by 25 W until exhaustion.	Ibuprofen: ↑ I-AFBP and L:R

CHO, carbohydrate. HRR, heart rate reserve. IL-6, interleukin-6. I-FABP, intestinal fatty acid binding protein. L:R, lactulose to rhamnose ratio. LPS–LPB, lipopolysaccharide-binding protein. PRO, protein. RH, relative humidity. TT, time trial. Wmax, maximum workload. ↑, increase. ↓, decrease.
